# Erythema annulare centrifugum as presenting sign of activation of breast cancer*

**DOI:** 10.1590/abd1806-4841.20154785

**Published:** 2015

**Authors:** Ilteris Oguz Topal, Yunus Topal, Aytul Sargan, Hatice Duman, Sule Gungor, Ozgur Emek Kocaturk Goncu, Selver Ozekinci

**Affiliations:** 1 Okmeydani Training and Research Hospital, Department of Dermatology - Istanbul, Turkey; 2 Gaziosmanpaşa Taksim Training and Research Hospital, Department of General Surgery - Istanbul, Turkey; 3 Okmeydani Training and Research Hospital, Department of Pathology - Istanbul, Turkey

**Keywords:** Adenocarcinoma, Carcinoma, Erythema

## Abstract

Erythema annulare centrifugum is a figurate erythema of unknown etiology. It has been associated with many different entities, including infections, food allergy, drug reactions and malignant neoplasms. Herein, we report a case of erythema annulare centrifugum as presenting sign of activation of breastcancer.

To Editor,

A 58-year-old woman sought us due to erythematous lesions on her abdomen. The patient had been diagnosed with breast cancer 4 year earlier and breast-conserving surgery with radiotherapy was performed. Lesions had first appeared before the diagnosis of cancer, and finally disappeared after the treatment. The patient said that erythematous annular lesions had started several months ago again. Dermatological examination revealed erythematous annular plaques with central clearing on the abdomen (Figure 1). Histopathological examination of punch biopsy obtained from annular lesion showed epidermal spongiosis, perivascular lymphocytic infiltrates and edema in the dermis (Figure 2). Routine laboratory findings were within the normal ranges. She presented no known food or drug allergies. Clinical and histopathological features were consistent with erythema annulare centrifugum. We suspected the lesions might be a sign of activation of cancer and the patient was referred to the Department of Oncology. Further investigations were carried out. Supraclavicular lymph node metastasis was detected by positron emission tomography (Figure 3) and chemotherapy was initiated.

**Figure 1:**
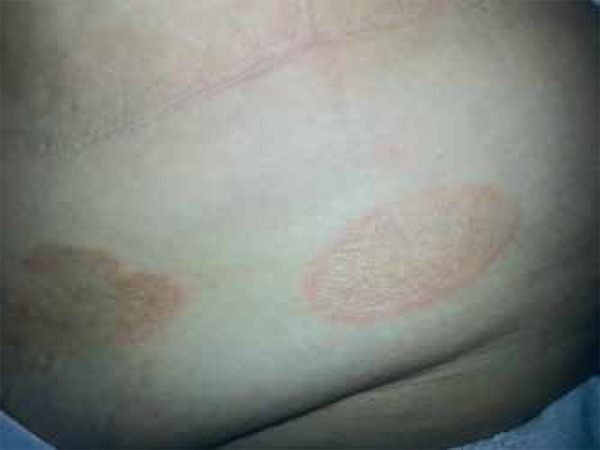
Annular, erythematous plaques with central clearing on abdomen

**Figure 2:**
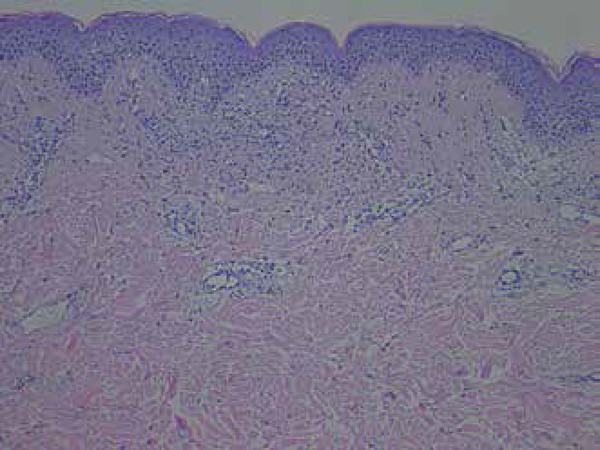
Epidermal spongiosis, perivascular lymphocytic infiltration and edema in dermis. (H&E,×40)

**Figure 3:**
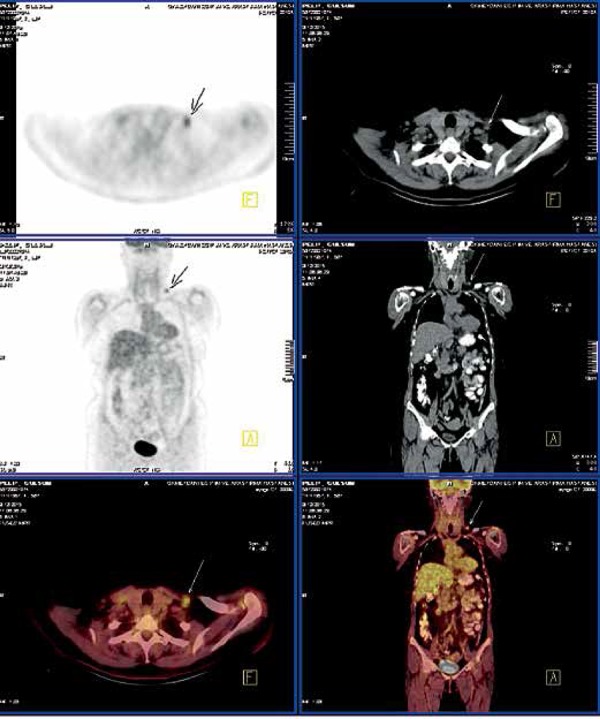
Positron emission tomography showing supraclavicular lymph node metastasis

Erythema annulare centrifugum (EAC) was first described by Darier in 1916. Clinically, the lesions are annular, or presented with raised borders, and they spread peripherally with a tendency of central clearing.^1^ Although its etiology is not known for certain, it is assumed to be a hypersensitivity reaction to malignancies, infections and drugs.^2^

EAC may be associated with an underlying malignancy. In these patients, lesions often appear and are resolved in a paraneoplastic manner. Annular lesions may follow, accompany or precede the malignancy.^3^ Kim et al conducted a clinicopathological analysis of 66 cases of EAC and found that 13% of the cases were associated with internal malignancies.^4^ Coexistence of EAC and breast cancer has been rarely reported.

Recently, Ko et al, reported a case of a 53-year-old woman who was diagnosed with EAC associated with breast cancer. The skin lesions appeared 3 weeks after surgery for breast cancer but the use of topical steroid and oral antihistaminics could not prevent the exacerbation of the lesions. The patient started chemotherapy and EAC disappeared dramatically with residual post-inflammatory pigmentation.^5^ In addition, Panasiti et al described a case of breast cancer presenting with EAC which was successfully treated by removal of the tumor.^2^

Another case has been reported by Dourmishev et al. In this case, annular lesions persisted for two months. Laboratory examinations were within the normal range. Concomitant bacterial and viral infections or systemic disease were not found. The authors considered the possible activation of the patient's previous breast cancer, but recurrence of malignancy was not detected.^1^ Similarly to this case, we suspected the possible activation of breast cancer. As result of further investigation, supraclavicular lymph node metastasis was detected.

The possible mechanisms of EAC association with malignancies are complex, but it suggested that the pathogenesis of EAC occurs through T helper cell (Th) 1 mediated reaction associated with some tumor factors such as tumor necrosis factor alpha and proinflammatory cytokines.^5^

In conclusion, EAC may be associated with malignancies. The detection of suspected paraneoplastic erythema annulare centrifugum is particularly important because it may enable the discovery of underlying cancer.

## References

[1] Dourmishev LA, Gergovska MJ, Nikolova KK, Balabanova MB (2010). Erythema Annulare Centrifugum in a Patient Operated on for
Breast Carcinoma. Acta Dermatovenerol Croat.

[2] Mir A, Terushkin V, Fischer M, Meehan S (2012). Erythema annulare centrifigum. Dermatol Online J.

[3] Chodkiewicz HM, Cohen PR (2012). Paraneoplastic erythemaannularecentrifugum eruption:
PEACE. Am J Clin Dermatol.

[4] Kim KJ, Chang SE, Choi JH, Sung KJ, Moon KC, Koh JK (2002). Clinicopathologic analysis of 66 cases of erythema annulare
centrifugum. J Dermatol.

[5] Ko WC, You WC (2011). Erythema annulare centrifugum developed post-breast cancer
surgery.JJ. Dermatol.

